# Angle grinder as surgical tool for incarcerated penoscrotal steel ring

**DOI:** 10.11604/pamj.2020.37.91.20784

**Published:** 2020-09-25

**Authors:** Dan Alin Pop, Bogdan Ioan Stanciu, Tudor Moisoiu, Dan Burghelea, Florin Ioan Elec

**Affiliations:** 1 Department of Transplantation, Clinical Institute of Urology and Renal Transplantation, Cluj-Napoca, Romania,; 2 Faculty of Medicine, Iuliu Hatieganu University of Medicine and Pharmacy, Cluj-Napoca, Romania

**Keywords:** Steel ring, penile strangulation, scrotal entrapment, angle grinder

## Abstract

Penile strangulation with concomitant scrotal entrapment by a steel ring is an extremely rare urological emergency that requires immediate intervention. Any delay may lead to irreversible complications. Metal rings increase penile engorgement and are usually associated with an attempt to improve sexual pleasure or to maintain a prolonged erection. The removal of steel rings can be challenging and may require a multidisciplinary approach. We present a unique case report of an 18-year-old male with a penoscrotal steel ring retained for 24 hours that was safely removed using an angle grinder as a surgical tool.

## Introduction

Penoscrotal strangulation by steel rings is an unusual urological condition that requires immediate intervention due to potentially severe complications. The use of metal rings is associated with an attempt to improve sexual performance and maintain a prolonged erection and has been described in adolescent boys and men [1]. In the pediatric population, this maneuver has been attempted to prevent enuresis [2]. Penile strangulation cause different degrees of vascular obstruction, from penile edema to ischemic necrosis and gangrene of tissues. In addition, a whole spectrum of mechanical penile injuries is recognized, such as skin ulcerations, loss of distal penile sensations, urethral injuries and development of urethral fistula [3]. Removal of steel rings can pose a difficult problem because of their characteristics and lack of adequate equipment available in hospitals. There are various techniques for extricating penile rings that have been described in the literature [3-6].

## Patient and observation

**Figure 1:**
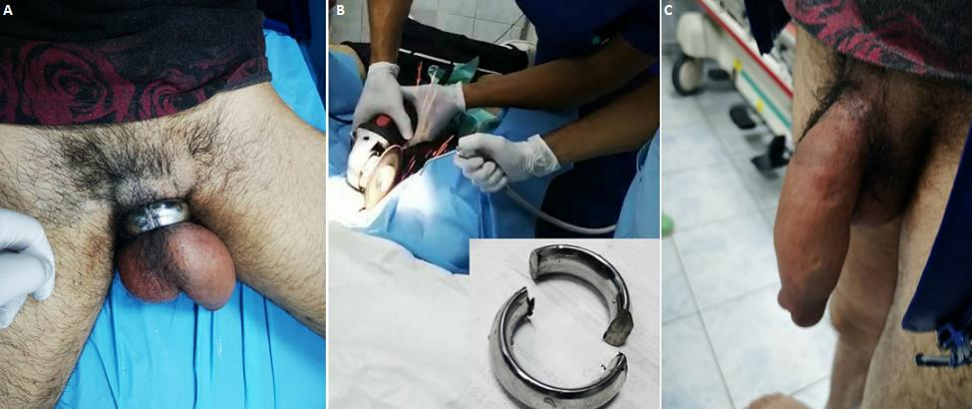
(A) penoscrotal strangulating steel ring; (B) angle grinder cutting the steel ring with saline solution cooling + disassembled ring (bottom right); (C) day 2 after ring removal

An 18-year-old man with no significant medical or psychiatric history presented to our emergency room with a painful swollen penis and scrotum. He had used a steel penoscrotal ring to improve sexual pleasure 24 hours before. The clinical examination found a steel ring (7.5cm in diameter, 2.4cm wide and 1.4cm thick) at the base of his penis and scrotum, with important penoscrotal edema and erythema over the strangulated area. Other physical examinations were normal. Laboratory results on admission were in normal range. Initial attempts to remove the ring with lubrication with Cathejell failed because of the distal edema with massive penile enlargement. Attempts to cut the ring with the help of a dental micromotor were also unsuccessfully due to the thickness and rigidity of the ring. The decision was then made to use an angle grinder (Heinner VPU007, 600 W, 12000 RPM, 115mm) to divide the steel ring, but due to its thickness, we were unable to remove it. The ring was successfully removed after cutting it on the other side with the same angle grinder. A wooden tongue depressor was inserted between the penis and the ring and cooling with a saline solution was performed. The operating time was about 18 minutes and no iatrogenic damage was noted. The outcome of the patient was uneventful, with edema resolution over the next two days (Figure 1).

## Discussion

Penile strangulation with concomitant scrotum entrapment is a rare and unusual urological emergency that must be resolved as soon as possible to avoid severe vascular and mechanical complications [3]. However, the majority of patients do not seek medical advice early because of the embarrassment. The practice of using constricting metal rings in adults is an attempt to achieve erotic goals, to improve sexual pleasure and prolong the erection. The management of this condition might be challenging for surgeons due to the variety of materials used and because of the inadequate equipment available. Bhat *et al*. have developed a simple classification of trauma by strangulation of the penis [3].

*Grade I:* edema of the distal penis, no evidence of skin ulceration or urethral injury.

*Grade II:* injury to the skin and constriction of corpus spongiosum but no evidence of urethral injury, distal penile edema with decreased penile sensation.

*Grade III:* injury to the skin and urethra but no urethral fistula, loss of distal penile sensation.

*Grade IV:* complete division of corpus spongiosum leading to urethral fistula and constriction of corpus cavernosum with the loss of distal penile sensation.

*Grade V:* gangrene, necrosis, or complete amputation of the distal penis.

There is no standard protocol for removing a strangulating penoscrotal ring, the choice of method depending upon trauma grade, incarceration time, type of material, and equipment availability. Initially, it was attempted to remove the ring by lubricating the entire penoscrotal area with Cathejell and milking the penis, but due to the important penoscrotal edema, this could not be practiced successfully. Subsequently, it was enlisted the help of colleagues dentists who suggested the use of a dental micromotor to make a section at the level of the penoscrotal ring. Five attempts were made to divide the ring, all resulting in the destruction of the dental micromotors due to the thickness and hardness of the ring. Thus, unlike other cases where this technique was successfully applied [7], in our situation it was necessary to find another management solution. Considering the circumstances, usage of an angle grinder as a surgical tool was a necessity. Besides the ring thickness and hardness, incarceration time was an important criterion to be considered in choosing the way of treatment, so the choice of cutting the ring with an angle grinder for fast removal was made. This technique is not risk-free with penoscrotal lesions on account of steel overheating or angle grinder misusage being possible. A wooden tongue depressor was inserted between the penis and the steel ring and cooling with a saline solution was performed continuously to prevent iatrogenic tissue damage. The particularity of our case consists in the impossibility of using the classical methods for detaching the strangulating penoscrotal steel ring and the necessity to make use of an atypical tool such as the angle grinder as a surgical instrument to successfully liberate the patient genitalia.

## Conclusion

Penoscrotal strangulation is a challenging urological emergency that requires management in the first few hours. If left untreated, severe complications such as neurovascular damage and gangrene of the penis might occur. This case report presents the successful use of an angle grinder as a surgical tool to remove a penoscrotal strangulating steel ring in an 18-year-old man and highlights the unconventional ways of treatment that might be required by this condition. It is worth remembering that a DIY tool can be operated as a surgical instrument in such unusual and extreme circumstances.
